# Paramedic Understanding of Tension Pneumothorax and Needle Thoracostomy (NT) Site Selection

**DOI:** 10.7759/cureus.27013

**Published:** 2022-07-19

**Authors:** Jeffrey S Lubin, Joshua Knapp, Maude L Kettenmann

**Affiliations:** 1 Department of Public Health Sciences, Penn State College of Medicine, Hershey, USA; 2 Department of Emergency Medicine, Penn State Health Milton S. Hershey Medical Center, Hershey, USA; 3 Department of Emergency Medicine, Penn State Health Community Medical Group, Camp Hill, USA; 4 Department of Emergency Medicine, Gunderson Health System, La Crosse, USA

**Keywords:** emergent chest decompression, prehospital intervention, prehospital, paramedic education, tension pneumothorax, needle decompression

## Abstract

Introduction

Tension pneumothorax is an immediate threat to life. Treatment in the prehospital setting is usually achieved by needle thoracostomy (NT). Prehospital personnel are taught to perform NT, frequently in the second intercostal space (ICS) at the mid-clavicular line (MCL). Previous literature has suggested that emergency physicians have difficulty identifying this anatomic location correctly. We hypothesized that paramedics would also have difficulty accurately identifying the proper location for NT.

Methods

A prospective, observational study was performed to assess paramedic ability to identify the location for treatment with NT. Participants were recruited during a statewide Emergency Medical Services (EMS) conference. Subjects were asked the anatomic site for NT and asked to mark the site on a shirtless male volunteer. The site was copied onto a transparent sheet lined up against predetermined points on the volunteer’s chest. It was then compared against the correct location that had been identified using palpation, measuring tape, and ultrasound.

Results

29 paramedics participated, with 24 (83%) in practice for more than five years and 23 (79%) doing mostly or all 9-1-1 response. All subjects (100%) reported training in NT, although six (21%) had never performed a NT in the field. Nine paramedics (31%) recognized the second ICS at the MCL as the desired site for NT, with 12 (41%) specifying only the second ICS, 11 (38%) specifying second or third ICS, and six (21%) naming a different location (third, fourth, or fifth ICS). None (0%) of the 29 paramedics identified the exact second ICS MCL on the volunteer. Mean distance from the second ICS MCL was 1.37 cm (interquartile range (IQR): 0.7-1.90) in the medial-lateral direction and 2.43 cm in the superior-inferior direction (IQR: 1.10-3.70). Overall mean distance was 3.12 cm from the correct location (IQR: 1.90-4.50). Most commonly, the identified location was too inferior (93%). Allowing for a 2 cm radius from the correct position, eight (28%) approximated the correct placement. 25 (86%) were within a 5 cm radius.

Conclusion

In this study, paramedics had difficulty identifying the correct anatomic site for NT. EMS medical directors may need to rethink training or consider alternative techniques.

## Introduction

Tension pneumothorax is a life-threatening emergency that requires urgent management. Often due to a traumatic lung laceration or spontaneous rupture of a pulmonary bleb, a tension pneumothorax is the accumulation of air in the pleural space to the point of hemodynamic compromise. Fortunately, medical personnel can temporize this physiological decompensation. Indeed, tension pneumothorax has been identified as one of the most common causes of potentially preventable death in combat [1]. In the prehospital setting this is commonly achieved by needle thoracostomy (NT). Although much discussion and literature has focused on alternative sites [2-4], a common recommendation is to place the needle in the second intercostal space (ICS) at the mid-clavicular line (MCL) just superior to the rib to avoid the neurovascular bundle [5].

A study of 25 emergency medicine physicians found that while this landmark was verbalized by 88% of the participants, only 60% were able to correctly identify the second ICS MCL on a human volunteer, with 95% indicating a point medial to the MCL [6]. Similarly, a study of 25 United States (US) Navy hospital corpsmen found a misplacement rate of 82% in a cadaver model [7]. Since this potentially life-saving skill is performed in the prehospital civilian setting by paramedics, we attempted to assess the ability of paramedics to identify the location for NT. We hypothesized that paramedics would have a low level of accuracy in identifying the correct anatomic location for needle decompression.

This article was previously presented as an abstract at the National Association of EMS Physician Annual Meeting in January 2019.

## Materials and methods

A prospective observational study was performed to assess the ability of paramedics to recognize a tension pneumothorax and their ability to identify the location for treatment with NT. Subjects were recruited at Pennsylvania’s annual statewide Emergency Medical Services (EMS) conference. Demographic data, including years of EMS practice and environment of practice, calls per week, percentage of EMS vs transport calls, specific training in NT, certification in Prehospital Trauma Life Support (PHTLS), and the estimated number of NTs each participant had performed in the field were recorded.

Subjects were asked to create a list of signs and symptoms of a tension pneumothorax, what the anatomic site for needle decompression/thoracostomy is, and what alternative sites there are for NT.

The correct location for NT was preidentified on two similarly sized human male volunteers using measuring tape to identify the MCL and palpation and ultrasound to locate the second ICS. This point, the second ICS MCL, was then copied over to a transparent sheet to create a template.

Each participant was instructed to identify the site for NT with a pen mark on one of the shirtless volunteers. The pen mark was copied over to a transparent sheet lined up against predetermined points on the volunteer’s chest and subsequently removed. The template was placed over each participant’s sheet, and the distance between the two points was measured.

## Results

A cohort of 29 paramedics was studied (Table 1).

**Table 1:** Demographics of participants EMS: Emergency Medical Services; NT = Needle Thoracostomy; PHTLS = Prehospital Trauma Life Support

Years of Practice	Percentage
1-5	17.2
6-20	41.4
21-37	41.4
Calls per Week	
1-12	50
13-20	28.6
21-40	21.4
EMS vs. Transport	
Most/all EMS	79.3
Equal	17.2
Most transport	3.5
EMS Environment	
Urban	
Yes	55.2
No	44.8
Rural	
Yes	72.4
No	27.6
Air	
Yes	10.3
No	89.7
NT Training	
Yes	100
No	0
Current PHTLS Certification	
Yes	35.7
No	64.3
Ever PHTLS Certified	
Yes	57.1
No	42.9
# NT Performed in the Field	
0	20.7
1-4	31.0
5-20	48.3

Of the participants, 24 (83%) had been in practice for more than five years (range: 1-37 years), 14 (50%) were running more than 12 calls a week, and 23 (79%) were doing mostly or all EMS. All subjects (100%) reported training in NT, 10 (34%) were currently certified in PHTLS, and 16 (55%) had previously been PHTLS certified. Six (21%) had never performed a NT in the field, whereas 14 (48%) had performed five or more (range: 5-20).

The most commonly noted ways to identify the need for a NT were assessment of breath sounds (89%), shortness of breath (67%), tracheal deviation (56%), jugular venous distention (26%), abnormal vital signs (30%), and evaluation of the chest (15%).

When asked to name the preferred site for NT, nine paramedics (31%) gave textbook answers of second ICS MCL. Twelve (41%) specified the second ICS without mentioning the MCL, whereas an additional 11 (38%) specified second or third ICS, with six (21%) naming a different location (third, fourth, or fifth ICS). The MCL was specified by 21 (72%), with the remainder not specifying except one person who named the mid-axillary line. Five (17%) noted that it should be performed just superior to the rib, with the others did not make any specification.

When asked to describe an alternative site for NT, five people provided no answer or an inadequate answer (e.g., “lateral chest”). Of the remaining, 23 (96%) specified anterior or mid-axillary line and 17 (71%) stated the fourth or fourth-to-fifth ICS. Three (13%) named the fifth-to-sixth or seventh ICS, and one (4%) named the second ICS at the mid-axillary line.

**Figure 1:**
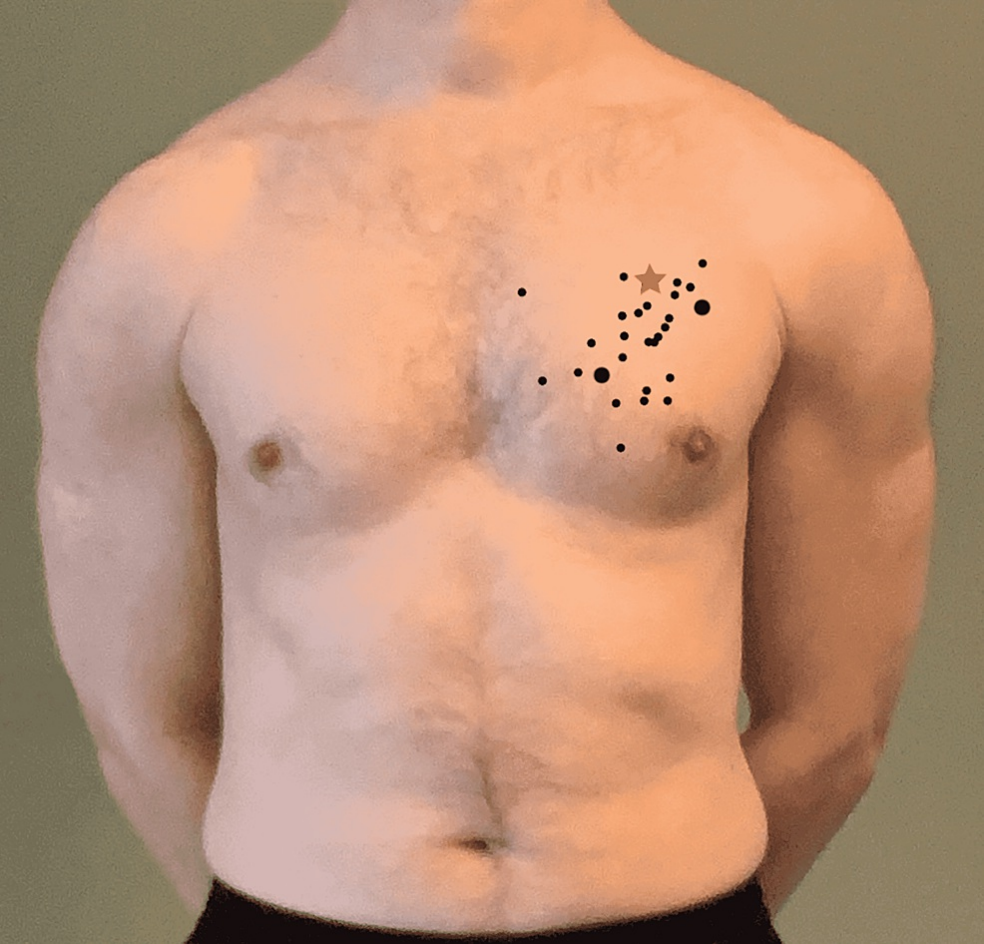
Range of points marked on the chest Star indicates the exact location of the second intercostal space (ICS) at the mid-clavicular line (MCL). Larger dots represent two subjects selecting the same point.

None (0%) of the 29 paramedics correctly identified the second ICS MCL on one of the volunteers, as summarized in Figure 1.

Allowing for a range of 0.5 cm superior (within the second ICS but not just below the third rib), one subject (3%) identified the second ICS. The identified location was too inferior for 27 (93%). Allowing for a range of 0.5 cm medial and 0.5 cm lateral of the MCL, 16 paramedics (55%) approximated the MCL. The identified location was too medial for 15 (51%) and too lateral for 14 (48%). Mean distance from the second ICS MCL was 1.37 cm (interquartile range (IQR): 0.7-1.90) in the medial-lateral direction and 2.43 cm in the superior-inferior direction (IQR: 1.10-3.70). Overall mean distance was 3.12 cm from the correct location (IQR: 1.90-4.50).

## Discussion

Compromising ventilation and circulation, tension pneumothorax is a threat to life, and addressing it is a priority in the critically ill or injured patient. This is done through needle decompression of the chest, converting a potentially deadly tension pneumothorax into a manageable open pneumothorax. The primary aim of this study was to evaluate paramedic knowledge of this entity, assess their ability to describe the preferred location for this procedure, and then determine if they could identify this point on a human volunteer. With tension pneumothorax occurring in an estimated one in 20 patients with major trauma [8] and 1-2% of 8,600 spontaneous pneumothoraces a year [9], this is a necessary skill for a paramedic to master. Indeed, according to the National EMS Scope of Practice Model, “minimum psychomotor skills of the Paramedic” include the ability to “decompress the pleural space” [10]. All of the paramedics in this study reported training in this skill. In this study group, 24 of the 29 (83%) had been in practice for more than five years. 79% of the participants had performed at least one NT in the field, and nearly half (48%) had performed more than five throughout their career.

The first aspect of mastering this skill is understanding when it is indicated. Paramedics in this study self-generated a list of signs and symptoms when asked how to diagnose a tension pneumothorax. Commonly listed symptoms of abnormal breath sounds, shortness of breath, tracheal deviation, and evaluation of the chest align with the Advanced Trauma Life Support (ATLS) dictum that “the presence of acute respiratory distress, subcutaneous emphysema, absent breath sounds, hyperresonance to percussion, and tracheal shift supports the diagnosis and warrants immediate thoracic decompression” [5]. Importantly, in one retrospective study from 2021 looking at 84 consecutive patients who had undergone NT in the field, 19% of the procedures performed appeared to have not been medically indicated [11].

The optimal location for NT continues to be debated in the literature [12,13]. Both the second ICS MCL and the fourth/fifth ICS in the anterior axillary line (ICS 4/5-AAL) have been proposed as the preferred locations. Although in 2018, the ATLS recommendations changed from ICS2-MCL to ICS4/5-AAL, the European Trauma Course (ETC) trauma guidelines and the guidelines from the Royal College of Surgeons of Edinburgh (RCSEd) in the UK still adhere to placement in the ICS2-MCL for the preferred location of NT. Potential complications that can arise from a poorly placed NT may include cardiac tamponade, life-threatening bleeding due to injury to the pulmonary artery or an intercostal vessel, and nerve injury at the insertion site [8]. The NT may also be nontherapeutic if not properly placed. With that in mind, only nine of the 29 paramedics (31%) provided an adequate answer when describing where they would perform a needle decompression. Many erroneously offered inferior points, with 11 (38%) suggesting that the second or third ICS were equivalent, and six (31%) stating the third, fourth, or fifth ICS. Subjects were more accurate at remembering the transverse landmark, with 21 (72%) correctly specifying the MCL. Interestingly, there was more accuracy [2,7,8] and consensus when providing alternative sites for NT, with the 25 of the 29 (86%) who specified a location describing the area of chest tube insertion, even though this procedure is less commonly performed by paramedics.

In a similar study published in 2021, paramedics were partnered and asked to identify the location for needle decompression on each other (both ICS2-MCL and ICS4/5-AAL). ICS2-MCL was correctly identified by 54 of 68 (79.4%) and ICS4/5-AAL was correctly identified by 43 of 68 (71.7%) participants. While this group of paramedics was somewhat more accurate than in our study, the study protocol called for a board-certified or board-eligible emergency medicine physician to confirm the location accuracy [14]. The literature suggests that physicians also have difficulty identifying the correct anatomic site for the procedure. In the group of emergency medicine physicians studied by Ferrie, Collum, and McGovern [6], there was a significant discrepancy between being able to cite the correct landmark for NT and the ability to identify it on a human volunteer. While all but one of Ferrie, Collum, and McGovern's [6] subjects went medial to the MCL, our study group was nearly split with 15 (51%) too medial and 14 (48%) too lateral, but with 16 (55%) within 1 cm of the MCL. In the longitudinal direction, our study group’s identified area was overall too inferior, with only one paramedic identifying the second ICS. This corresponds to the more inferior targets the study group cited as the preferred anatomic location (i.e., they identified an inferior location because they were aiming for an inferior location). Overall, mean distance from the preferred site was 3.12 cm, with a range of 1.1 to 6.6 cm. With the proximity of the intercostal neurovascular bundle, lung parenchyma, subclavian vessels, and heart, this inaccuracy is highly concerning for the potential for iatrogenic injury [8].

While potentially lifesaving, the invasive and emergent nature of NT has made it a controversial procedure. Studies have looked at both its efficacy [11,15] and its safety [8,16] with proposals for different locations [7,12], equipment [17-19], and abandoning it all together in favor of finger thoracostomy [20]. This study adds to the impetus to improve upon the procedure by suggesting the preferred site for NT is simply difficult to find. As with the 25 emergency medicine physicians studied who had at least an 85% misplacement rate [6] and the 25 US Navy Hospital corpsmen-who had just prior undergone a standardized training session-who had a misplacement rate of 82% [7], the 29 paramedics in this study had at least a 97% misplacement rate, with only one identifying the second ICS.

Limitations

The limitations of this study include a small sample size of paramedics. Furthermore, these paramedics all practiced in Pennsylvania, had self-selected to attend the annual EMS Conference, and volunteered for this study, leading to possible selection bias. More diverse subjects with a large number of randomly selected geographically diverse paramedics may lead to data more representative of paramedics as a whole. Although spontaneous tension pneumothorax occurs more commonly in men [21] and trauma victims are more often male, a second limitation is the use of only male models with normal body mass indices. Male models were chosen due to modesty concerns in the public venue of the conference hall, but minimized the difficulty in identifying thoracic surface anatomy as obscured by mammary or other adipose tissue.

## Conclusions

Despite acknowledging training in NT, a procedure reportedly done on patients in the field by many of the paramedics in this study, participants cited and identified appropriate locations for NT with low frequency. This study, along with many other studies, notes that NT is often done in incorrect patients, at incorrect anatomical locations, and with suboptimal success. With ongoing controversy regarding the best practice regarding NT, it is a prime time to readdress how paramedics are trained in this procedure. Attention to this crucial prehospital procedure could mean the difference between a patient who survives to the hospital and one who does not.
